# Usefulness of robot‐assisted radical prostatectomy in a patient with oligometastatic castration‐resistant prostate cancer

**DOI:** 10.1002/iju5.12229

**Published:** 2020-10-08

**Authors:** Takuya Koie, Noriyasu Hagiwara, Toru Yamada, Hiromi Kondo, Hiroki Ito, Masayuki Tomioka, Keisuke Kawashima, Daiki Kato, Koji Iinuma, Keita Nakane

**Affiliations:** ^1^ Department of Urology Gifu University Graduate School of Medicine Gifu Japan; ^2^ Department of Urology Matasunami General Hospital Hashima‐gun Japan; ^3^ Department of Urology Tokai Central Hospital Kakamigahara Japan; ^4^ Department of Pathology and Translational Research Gifu University Graduate School of Medicine Gifu Japan

**Keywords:** bone metastasis, castration‐resistant prostate cancer, oligometastasis, robot‐assisted radical prostatectomy

## Abstract

**Introduction:**

The patients with prostate cancer and low‐volume osseous metastases who underwent local definitive therapies had lower risks of cancer‐specific mortality. The usefulness of local definitive therapy for metastatic prostate cancer remains unclear.

**Case presentation:**

A 76‐year‐old man visited a private hospital with a chief complaint of left lower limb pain. His serum prostate‐specific antigen level was 365.156 ng/mL. Histological evaluation led to the initial diagnosis of adenocarcinoma of Gleason score 4 + 4 and clinical stage T3a N1 M1b. Although androgen deprivation therapy was performed, he developed metastatic castration‐resistant prostate cancer 6 months after the initial treatment. Therefore, he received enzalutamide and attained a serum prostate‐specific antigen level of 0.002 ng/mL 7 months after the second treatment. We performed robot‐assisted radical prostatectomy 1 year after diagnosis. Histopathological examination revealed that prostate cancer cells disappeared into the prostate.

**Conclusion:**

Robot‐assisted radical prostatectomy in selected patients with metastatic castration‐resistant prostate cancer may improve oncological outcomes.

**Abbreviations & Acronyms**
ADT: androgen deprivation therapy
BT: brachytherapy
CSM: cancer‐specific mortality
CSS: cancer‐specific survival
IQR: interquartile range
mCRPC: metastatic castration‐resistant prostate cancer
mPCa: metastatic prostate cancer
MRI: magnetic resonance imaging
OS: overall survival
PCa: prostate cancer
pCR: pathological complete response
PLND: pelvic lymph node dissection
PSA: prostate‐specific antigen
RARP: robot‐assisted radical prostatectomy
RP: radical prostatectomy

**Keynote message**

RARP may be performed safely in patients with castration‐resistant PCa and low‐volume osseous metastases.

## Introduction

Treatment options for mPCa have significantly evolved recently.^1^ Despite several new therapeutic agents with proven survival benefit for mCRPC, oncological outcomes have remained poor, with a 5‐year relative survival of 30% in patients with mPCa.^1^ However, patients with PCa and low‐volume osseous metastases who underwent local definitive therapies, including RP or BT, had lower risks of CSM than those who did not.^2^ Although the usefulness of local definitive therapy for mPCa remains unclear, RP may improve oncological outcomes in selected patients with mCRPC. Herein, we report the case of a patient with mCRPC treated with RARP who attained pCR after ADT.

## Case presentation

**Fig. 1:**
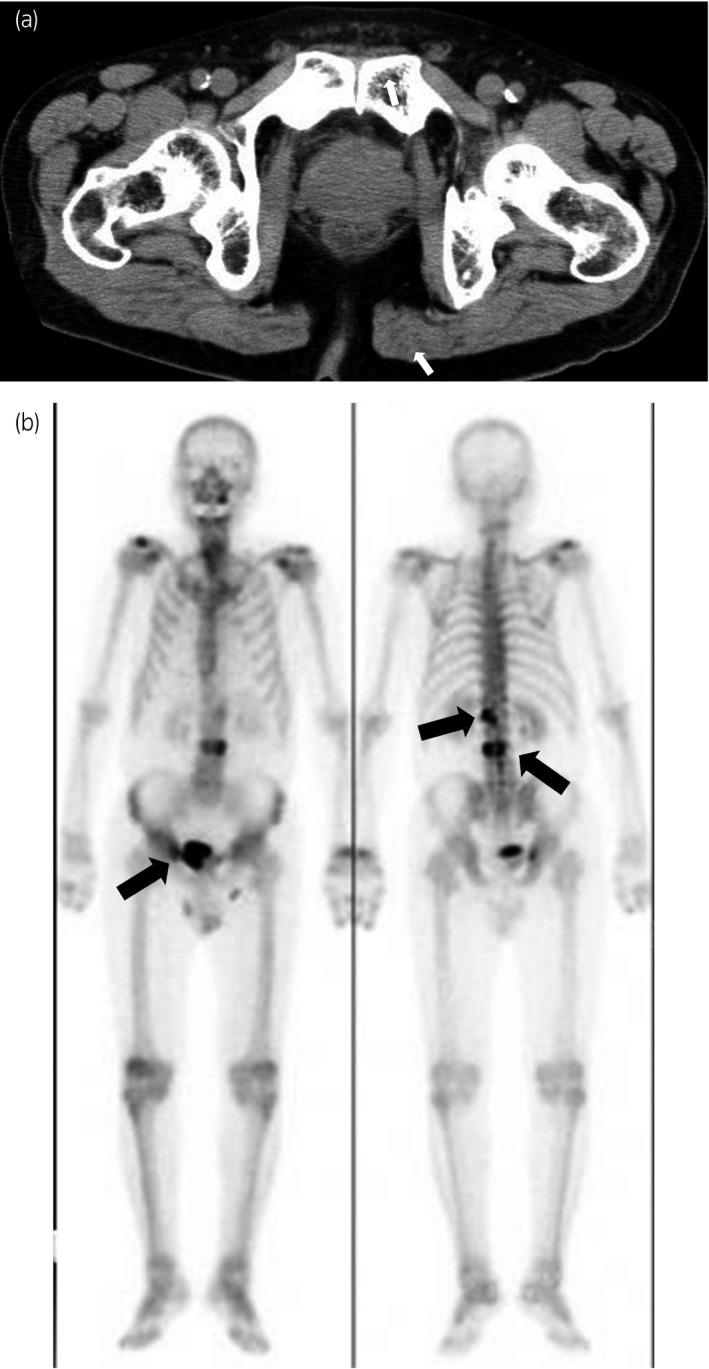
(a) Computed tomography image showing an irregular enlarged prostate in the left lobe (arrows). (b) Bone scintigraphy image showing multiple bone metastases (arrows).

**Fig. 2:**
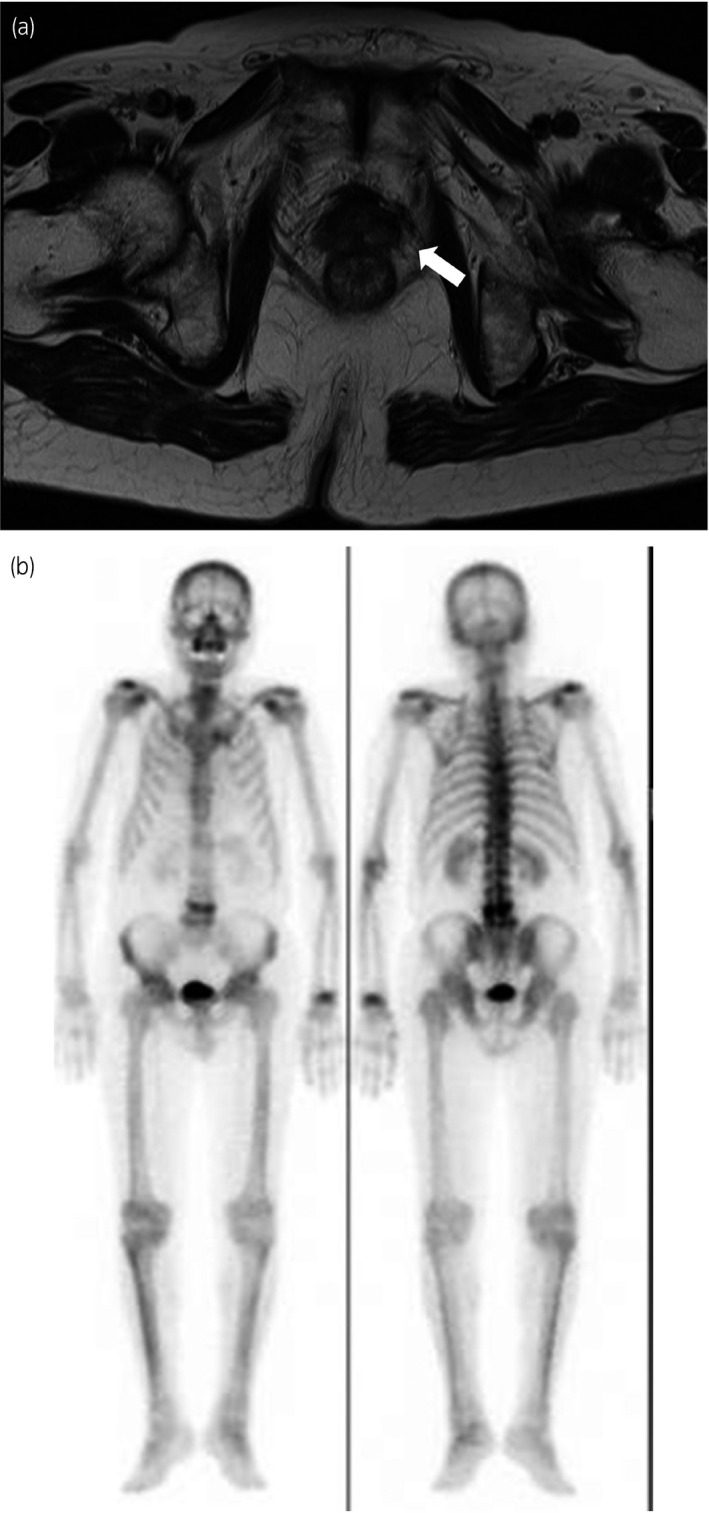
(a) Magnetic resonance image shows a clearly decreased prostate volume (arrows). (b) Bone scintigraphy image shows that the multiple bone metastases had disappeared.

**Fig. 3:**
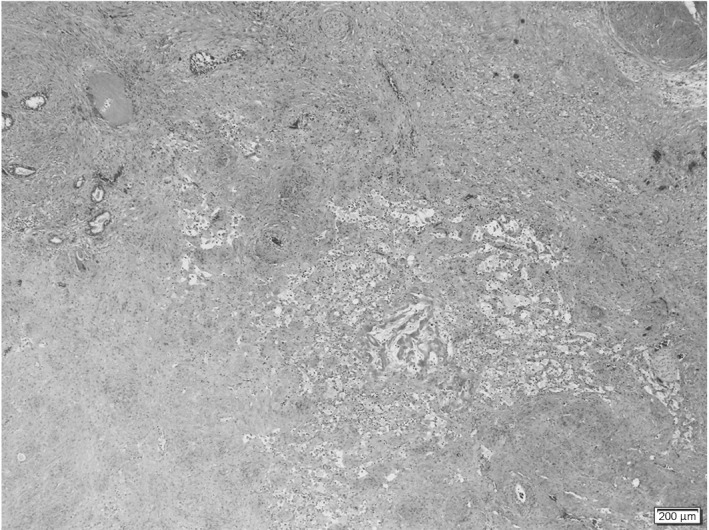
Histopathological findings showing no evidence of prostate cancer (hematoxylin and eosin staining).

A 76‐year‐old man visited a private hospital with a chief complaint of left lower limb pain. Computed tomography and MRI revealed an irregular enlarged prostate in the left lobe, left obturator lymph node involvement, and multiple bone metastases (Fig. 1a). Bone scintigraphy confirmed the multiple bone metastases (Fig. 1b). A transrectal prostate biopsy was performed, as his serum PSA level was 365.156 ng/mL (normal range: <4.0 ng/mL). Histological evaluation led to the initial diagnosis of adenocarcinoma of Gleason score 4 + 4 and clinical stage T3a N1 M1b. ADT was performed immediately. The nadir PSA level was 0.213 ng/mL 13 months after ADT, the obturator lymph node was unclear but gradually increased. He developed CRPC 6 months after the initial treatment. Therefore, he received enzalutamide 160 mg/day and attained a serum PSA level of 0.002 ng/mL 7 months after the second treatment. The prostate volume was clearly decreased on MRI (Fig. 2a). The multiple bone metastases had disappeared on bone scintigraphy (Fig. 2b). As we deemed the patients’ mCRPC to be curable with immediate surgery, we performed RARP without PLND 1 year after diagnosis. The console time was 54 min, and the estimated blood loss was 10 mL. No surgery‐related adverse events occurred. Histopathological examination revealed that PCa cells disappeared into the prostate, and he attained pCR (Fig. 3). He maintained an undetectable serum PSA level 6 months after surgery.

## Discussion

The curative treatment of oligometastatic PCa is likely to require a three‐tiered approach, including local consolidative therapy of the primary tumor, metastasis‐directed therapy, and systemic chemohormonal therapy.^2^ The ultimate goal of such an approach is to improve survival in patients with mPCa. In particular, hormone‐naïve mPCa should be treated with ADT and/or docetaxel or abiraterone acetate and prednisone therapies.^3^ More recently, the feasibility of cytoreductive RP and the benefit in terms of time to CRPC onset, OS, and frequency of local PCa progression with lower and upper urinary tract obstructions.^3^ Several retrospective studies using data from the Surveillance, Epidemiology, and End Results database assessed OS and CSS in patients who underwent local therapies, including RP or BT, in comparison with those who did not.^2^, ^3^ In the multivariate risk regression analysis, the patients with RP or BT had 62% and 32% decreased risks of CSM, respectively.^2^ In addition, Heidenreich *et al*. showed the benefits of cytoreductive RP in patients with ≤3 bone metastases, no visceral or extended lymph node metastases, and response to ADT (CRP group) in comparison with those in patients who received ADT alone (control group).^4^ Although OS was similar between the groups, radiographic progression‐free and CSS were significantly better in the CRP group (*P* = 0.003 *vs* *P* = 0.004).^4^

From the “seed and soil” theory, a receptive microenvironment (the “soil”) is required, into which disseminating cancer cells (the “seeds”) can engraft and form metastases.^2^ Therefore, the proposed mechanisms of benefit include elimination of the immunosuppressive effect of the primary tumor, removal of the source of lethal clone reseeding and systemic release, and avoidance of local progression morbidity.^2^, ^5^ The concept of cytoreductive surgery is well established and beneficial with regard to oncological outcomes in many other cancers, including ovarian, colon, and renal cell carcinomas.^1^ Conversely, several studies have demonstrated that the benefit of local treatment is directly linked to the risk of CSM.^2^

In this case, PLND was not performed during the RARP. For this reason, we consider that the patients may potentially have several invisible metastases at surgery. Currently, the European Association of Urology guideline recommends RP with extended PLND for high‐risk or node‐positive PCa as an optional treatment.^6^ Sooriakumaran *et al*. reported the perioperative outcomes for PCa patients with distant metastases who underwent RP.^7^ The median number of lymph nodes removed was 18 (IQR 11–27) and 71.7% of the patients had lymph node involvement.^7^ Likewise, 28.6% of the patients had pathologically node‐negative disease, even though those who underwent PLND with median removal of 17 lymph nodes (IQR 11–25).^5^ Conversely, patients with low‐volume bone metastases who underwent cytoreductive RP with PLND did not have a positive effect with respect to oncological outcomes, including overall and castration‐resistant free survival, than those who received best systemic therapy.^8^ Therefore, the efficacy of cytoreductive RP and PLND in patients with mCRPC remains unclear, and it is difficult to identify whether definitive therapies for the primary site should be performed for these patients.

In this case, the follow‐up period was relatively short. Therefore, long‐term follow‐up period is needed to decide the utility of RARP in this patient. The available data from several retrospective studies suggest that local therapies for PCa can be performed safely in patients with mPCa. However, data are insufficient to draw conclusions regarding the effect of consolidative therapies, including RP, on OS or CSS. Further studies are warranted to improve oncological outcomes in patients with mPCa, especially mCRPC, who underwent cytoreductive prostatectomy.

## Conflict of interest

The authors declare no conflict of interest.
